# Hidden Harm—Exploring the Utility of Geostatistical Analysis to Identify Child Criminal Exploitation (CCE)

**DOI:** 10.3390/bs16040613

**Published:** 2026-04-20

**Authors:** Antoinette Keaney-Bell, Colm Walsh

**Affiliations:** School of Social Sciences, Education and Social Work, Faculty of Arts, Humanities and Social Sciences, Queen’s University Belfast, Belfast BT7 1NN, UK; colm.walsh@qub.ac.uk

**Keywords:** geostatistics, Child Criminal Exploitation (CCE), Getis-Ord Gi*, Anselin Local Illicit networks, Moran’s I, network disruption, prevention, violence

## Abstract

This interdisciplinary study integrates criminological theory with geospatial methods to analyse large, multi-format datasets using geostatistical techniques. The aim is to predict where Child Criminal Exploitation (CCE) is likely to cluster, based on the spatial convergence of contextual risk factors. Drawing on insights from General Strain Theory (GST) and prior research on CCE, this study integrated seven open-source datasets capturing educational attainment, age demographics, violent crime, deprivation, and paramilitary-related violence. These variables were operationalised to construct a proxy measure for strain. Spatial analysis was conducted using ArcGIS Pro, including the Data Interoperability extension, to enable efficient integration and interrogation of multi-format geospatial data. Geospatial analysis demonstrated that contextual risk factors for CCE are spatially clustered. Using four search parameters, a small subset of wards with elevated risk were identified. This resulted in a reduction in ward locations by 85–99%, land area under investigation from 14.45% to 0.84%, and affected population from 17.91% to 1.41%, enabling more targeted and efficient resource allocation. As understanding of the contextual factors contributing to CCE improves, this methodological approach offers scalable and data-driven means of identifying high-risk areas. By integrating geospatial analysis with criminological theory, the model supports more effective safeguarding strategies and prioritisation of limited public resources. This study is limited by the absence of multi-agency datasets, which were beyond its scope. Future research aims to incorporate cross-sector data to validate and refine the model through ground-truthing, enhancing its predictive accuracy and practical applicability.

## 1. Introduction

### 1.1. Child Criminal Exploitation (CCE)

Child Criminal Exploitation (CCE) refers to the coercion, control, and/or manipulation of children into criminal activities by individuals or groups exploiting power imbalances. Despite commitments under the UN Convention on the Rights of the Child (UNCRC) and Sustainable Development Goals (SDGs) to reduce violence by 2030 (UNICEF, 2024; Walsh, 2024), exposure to this form of violence remains a challenge. CCE manifests in various forms, including drug supply (Moyle, 2019; Windle et al., 2020), violence via off/online grooming, threat of physical violence and coercion through interpersonal relationships and/or criminal networks (Naughton et al., 2020; Naughton & Redmond, 2020; and O’Meara Daly et al., 2020), through, for example, indebtedness to exploitative others (Walsh, 2023; Robinson et al., 2019), and involvement in civil unrest and violent public disorder (Walsh, 2021). Although research has shown that individual-level risk factors play a significant role as push/pull factors that elevate the risk of CCE (Moyle, 2019), at the heart of the issue remains the contexts that exacerbate these risks (Firmin & Lloyd, 2023).

Recent research has increasingly examined the broader contextual factors that heighten children’s vulnerability to criminal exploitation. These factors may be harmful in isolation, but when they co-occur in multiples, they significantly amplify the risk of criminal harms such as CCE. This exploratory analysis identifies and maps both established and emerging priority areas to help determine whether further evaluation is warranted and where such efforts should be most effectively targeted.

### 1.2. CCE in Context

CCE has emerged as a significant policy concern across the United Kingdom and Ireland over the past decade (Brewster et al., 2021; Department of Children and Youth Affairs, 2016; Naughton et al., 2020; Naughton & Redmond, 2020; and O’Meara Daly et al., 2020). Despite this, academic research remains limited (Maxwell et al., 2019, p. 18). In the absence of dedicated legislation, CCE is addressed under broader legal frameworks, such as the Modern Slavery Act 2015 (UK Parliament, 2015), the Children and Young Persons Act 1933, the Child Abduction Act 1984 and the Children Act 1989 (Maxwell et al., 2019, p. 8). Although advocacy groups have called for statutory recognition (The Children’s Society, 2021; Brown, 2022), CCE still lacks a formal statutory definition in UK legislation.

In Northern Ireland, the Department of Justice (Northern Ireland Executive, 2024) defines CCE as “a form of child abuse which occurs where an individual or group takes advantage of an imbalance of power to coerce, control, manipulate or deceive a child or young person under the age of 18 into any criminal activity. The exploitation may be through violence or the threat of violence, but may also appear to be transactional and in the context of perceived relationships and friendships. The victim may have been criminally exploited even if the activity appears to be consensual.” In this context, empirical research has shown that CCE may occur without physical contact and can involve technology and social media (Walsh, 2023). Common forms include drug storage or distribution, weapon holding, theft, violence, intimidation, vandalism, forced labour, and other criminal activities, often facilitated through grooming by trusted individuals. Recent legislation in the Republic of Ireland focused on grooming of children into crime (Department of Justice, 2024). Despite slow progress, the existing corpus of evidence is enhancing our understanding of the factors that contribute to and sustain CCE; however, to date, that evidence has not been sufficiently well embedded into practical tools that can help to identify where known risks are concentrated.

Northern Ireland may be often seen as synonymous with war, following the longest-running conflict on European soil. Over decades, paramilitaries, which were traditionally thought to be structured to engage in that war, have provided the networks that have facilitated the criminal exploitation of children as the conflict has transitioned towards peace. While 1998 was a watershed moment for society in the north of Ireland, recent prevalence data show that even those who were born post-1998 continue to be affected in various ways by these criminal networks (Walsh et al., 2025a). This is supported by McCalmont (2024), who estimated that paramilitary-linked violence affects 40% of adults and 45% of young people, with ongoing recruitment into criminal activities. Walsh (2025) documents the lives of children, young people and adults who are affected in different ways by paramilitaries in contemporary Northern Ireland. One of the most illuminating insights is that paramilitary structures now reflect what many would refer to as organised criminal gangs (OCGs) in other contexts. Indeed, in an insightful study capturing young people’s experiences of CCE, Walsh (2021) found that while business models vary from place to place, the phenomenon of CCE was intimately connected to, and even operationalised through, paramilitary structures in the NI context. While we are coming to understand these ‘business models’ of exploitation, and the contexts that induce them, we also know that they are under-evaluated.

### 1.3. Contextual Harm

Seeking to shift the emphasis and responsibility for exploitation away from individuals, Walsh (2023) found that CCE is nested in contexts that were themselves harmful. He coined the phrase ‘contextual harms’ as a way to represent the multi-level vulnerabilities that compound in multiples to elevate and even induce the risk of CCE. These contextual harms were defined as:

*…the presence and cumulative impact of a range of risk-related structural and demographic (e.g., poverty, deprivation, ethnicity and gender), social (e.g., family functioning, placement stability, lack of social support, and exposure to violence), psychological (e.g., psychological stress and lack of evidence-based treatment) and educational factors (e.g., truancy and academic attainment), that collectively contribute towards criminal harms*.

(Walsh, 2023, pp. 284–285)

This concept of contextual harm is derived from observations in prevalence data (i.e., the characteristics of communities where CCE is likely to occur) and qualitative data (how children, young people and professionals describe the most salient vulnerabilities). While this could provide very useful and practical insights into where CCE is likely to be elevated, and where resources could be better targeted, there have, however, been challenges with operationalising this idea of contextual harm. This paper presents one dimension of a wider programme of research. The broader study (Walsh et al., 2025b) examined how local statutory systems currently understand and respond to CCE and was conducted as part of a strategic review of regional responses to CCE. The datasets were analysed with two core objectives: (a) to capture the experiences of professionals working within sectors relevant to CCE, and (b) to assess the geographical areas in which CCE is likely to cluster. This paper reports specifically on findings related to objective (b), focusing on identifying and assessing the geographical areas where CCE is most likely to cluster.

### 1.4. Theorising Contextual Harm

Contextual harms may be conceived of as the conditions that induce strain (Agnew, 1992). Thus, the idea of ‘contextual strains’ (Walsh, 2023) is a helpful heuristic, consistent with orthodox criminological perspectives, such as General Strain Theory (GST), which posits that criminal behaviour arises when individuals experience strain and lack legitimate coping mechanisms. Agnew (2013, p. 654) emphasises that ‘criminal coping’ is most likely when specific personal and contextual factors converge. This convergence is central to understanding CCE, which is often exacerbated by overlapping structural harms, such as deprivation and violence. While criminological studies frequently isolate the conditioning variables, they often overlook the broader environmental context in which strain occurs, but place-based analyses are not alien to criminologists.

Criminological research has frequently demonstrated that crime is spatially concentrated, often occurring within micro-geographic units such as street segments or neighbourhoods (Weisburd & Amram, 2014). Rooted in the Chicago School, the spatial dimension of crime has long been a focus of criminological inquiry. Dong et al. (2020, p. 801) observe that “approximately 5% of street segments account for 50% of total crime”, underscoring the utility of crime mapping in prevention strategies (Hall & Yarwood, 2024).

Emerging evidence suggests that CCE similarly manifests at the community level, shaped by localised vulnerabilities (Maxwell & Wallace, 2021). Despite the relevance and utility of transdisciplinary and geographical approaches—such as environmental determinism (Livingstone, 2011)—criminology has yet to fully integrate spatial analysis into its frameworks. Weisburd (2015) advocates for a shift toward micro-geographic criminology, particularly in relation to hot spot analysis, arguing that “there is much room to make new discoveries and to examine new problems” (Weisburd, 2015, p. 150).

This study responds by incorporating Geographical Information Systems (GISs) into criminological analysis, extending General Strain Theory (Agnew, 1992, 2013) beyond individual-level stressors that precipitate criminal coping, to include spatially embedded ‘contextual strains’. These refer to geographically concentrated stressors—such as deprivation, exposure to violence and paramilitary activity—that shape young people’s lived experiences. Tobler’s (1970) First Law of Geography, which posits that proximate phenomena are more interrelated than distant ones, supports the notion that strain is not randomly distributed but spatially patterned. Through this lens, neighbourhoods with overlapping structural disadvantages create environments where strain is both intensified and normalised, increasing the likelihood of criminal coping. By integrating geostatistical methods with criminological theory, this study proposes a place-based extension of strain theory, using spatial clustering of risk to identify elevated vulnerability to CCE. Five contextual strains were selected to explore this broader environmental context.

#### 1.4.1. Contextual Strain 1: Educational Under-Attainment

School exclusion is a key risk factor for youth offending, violence and criminal exploitation, and while there is no shortage of studies drawing associational links (e.g., Arnez & Condry, 2021; Briggs, 2010) and proposed mechanisms (e.g., Sanders et al., 2018), the more recent literature has sought to estimate causal effects. In their experimental study, Cathro et al. (2023) found that school exclusion was causally linked to youth violence.

While exclusion for behaviours such as drug or weapon possession is often deemed necessary (Temple, 2020), Dando et al. (2022) argue that such behaviours may signal criminal exploitation (CCE). Emerging evidence suggests that some exclusions may be orchestrated by exploiters to further victimisation (Temple, 2020). Arnez and Condry (2021, p. 23) caution against attributing direct causality, instead framing exclusions within a broader system of “accumulated disadvantage”. Walsh (2021), however, went further, arguing that exclusion was not only a potential signal of exploitation but could elevate the risk further. Drawing on the concept of ‘safe spaces’, Walsh suggested that the more such spaces are reduced, the greater the pull towards exploitative ‘others’.

#### 1.4.2. Contextual Strain 2: High Youth Density

CCE is an age-dependent phenomenon, involving the manipulation or coercion of children under 18 into criminal activity, often through county lines supply chains, forced acquisitive crime or ancillary roles (Norman, 2024; Maxwell, 2024). While all children are at risk, those lacking protective structures are more vulnerable (Norman, 2024). High youth density represents one factor that, when combined with other factors, could be conceived of as a contextual strain relevant to the challenge of CCE. Areas with larger youth populations face elevated exploitation risks, particularly in areas where poverty (Firmin & Lloyd, 2023), school exclusion (Craig et al., 2017) and normalised violence intersect (Fowler et al., 2009). Marshall (2022) challenges passive victim narratives, suggesting that youth involvement in exploitative work may reflect responses to socioeconomic marginalisation. The cumulative impact of these harms can push vulnerable young people toward criminal opportunities, especially when adults manipulate such vulnerabilities (Walsh, 2023). Logically, if CCE is age-dependent, then a higher proportion of young people in a given context elevates the risk of CCE.

#### 1.4.3. Contextual Strain 3: Deprivation

Deprivation has long been implicated in criminality; however, research on CCE conceptualises economic and material deprivation as a lever pulled by exploiters for criminal gain (Whittaker et al., 2019). Harding (2020) argues that deprivation creates a broader ‘pool of availability’ for recruiters, with young people perceiving illicit income-generating activities—such as drug dealing—as a means of escape from hardship, despite the heightened risk of violence and arrest. The strain of deprivation can obscure victims’ understanding of their circumstances, and it is common for them not to recognise their experiences as being harmful (Apland et al., 2017; Robinson et al., 2019; Maxwell, 2024).

#### 1.4.4. Contextual Strains 4 and 5: Exposure to Paramilitary Activity and Elevated Violent Crime in Northern Ireland

When children and young people are exposed to violence, violence becomes normalised (Fowler et al., 2009). In violent contexts, violence becomes increasingly more probable. Given that CCE itself is a manifestation of violence—both symbolic (Bourdieu, 1989) and instrumental (Stuart & Stuart, 2020) violence itself becomes part of a context that is harmful. Violence becomes the fulcrum, itself causing harm and, in turn, this harm elevating the risk of further violence. The cumulative impact of these harms may compel vulnerable young people towards criminal opportunities in their communities, particularly when such vulnerabilities are leveraged by adults (Walsh, 2023). Young people most affected by contextual harm are often those at greatest risk of criminal exploitation (Moyle, 2019; Robinson et al., 2019; Walsh, 2023). Crucially, these risk factors are not simply related with higher crime rates; rather, they are complex and intertwined with broader indicators of social and economic wellbeing (Firmin & Lloyd, 2023).

Exposure to violence—direct or indirect—is associated with a range of adverse psychological outcomes, including heightened stress responses (e.g., hypervigilance), maladaptive coping strategies (e.g., substance use), and aggression (Fowler et al., 2009). Among children in the justice system, those who have experienced violent victimisation are significantly more likely (nine times more likely) to be convicted of violent offences (Walsh, 2021). These findings align with longstanding evidence on the cycle of violence (Widom, 1989). Living in areas characterised by elevated levels of violence further amplifies vulnerability to criminal exploitation.

In Northern Ireland, this dynamic is shaped by the normalisation of violence in communities affected by paramilitary activity. Victims of violence are rendered vulnerable, and this vulnerability increases their susceptibility to exploitation. Paramilitary groups have been linked to CCE and contribute to environments in which violence is tolerated, expected, or embedded in local community life. This creates conditions that both obscure exploitation and heighten risk for young people.

Although post-Good Friday Agreement research suggested paramilitarism had shifted towards organised crime (Moran, 2004), recent political tensions surrounding immigration, Brexit, and the Irish Sea Border have reignited concerns about renewed violence (Walsh, 2021). Evidence indicates, however, that violence has remained embedded in some communities, shaping young people’s everyday lives (Hamill, 2011; Walsh, 2021, 2023). Paramilitary groups are not external actors but integral to local social structures—often neighbours or family members—creating persistent fear and insecurity (Walsh, 2021). Young people report being most affected in familiar spaces, despite feeling unsafe elsewhere (Walsh, 2021).

Exposure to such violence constitutes a significant contextual harm, influencing mental health through fear, paranoia, and maladaptive coping (McAlister et al., 2018). Violence is normalised as part of daily life and underpinned by coercive control (Hamill, 2011). CCE represents a specific manifestation, where young people are coerced into criminality for adult benefit, with implications for safety and wellbeing (Walsh, 2023). This normalisation risks tacit acceptance of victimisation, meaning children may not self-identify as victims and instead encounter punitive responses (Fowler et al., 2009; Robinson et al., 2019).

Recruitment into paramilitary groups persists, irrespective of political or criminal motivations (Walsh & Cunningham, 2019). Similar to hyperlocal CCE recruitment elsewhere in the UK (Harding, 2020), strategies in NI range from offering alcohol to requesting tasks such as burglary or arson (Walsh & Schubotz, 2019). Uniquely, recruitment often reflects generational patterns: parents involved in paramilitary activity encourage children’s participation (McAlister et al., 2018). This dynamic was evident during the 2021 NI riots, where familial involvement legitimised youth engagement (Walsh, 2021). Although organised crime groups prioritise profit, and paramilitaries project a priority of ideology, boundaries increasingly blur into hybrid organisations that combine political symbolism with organised criminality (Walsh & Cunningham, 2019; Northern Ireland Executive, 2024).

While NI serves as the focal point for this study, the GIS method is adaptable to other contexts, offering a scalable framework for identifying and analysing geographic clusters of CCE. GISs offer a powerful tool for analysing these complex phenomena, enabling the integration and spatial interrogation of diverse datasets, supporting the identification of high-risk areas. Silva and Oliveira (2022) emphasise its methodological versatility, particularly in bridging quantitative and qualitative approaches. To explore the relationship between violent crime and CCE, geostatistical analysis was conducted, testing the null hypothesis that the spatial distribution of violent crime in NI is the result of a random spatial process.

## 2. Methods

### 2.1. Design and Approach

**Figure 1:**
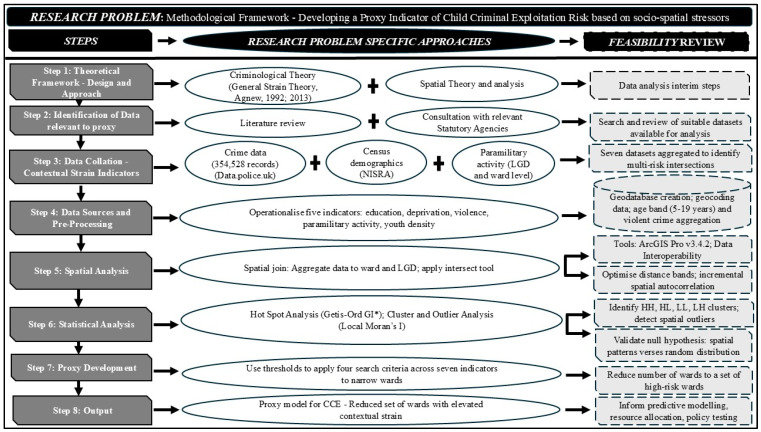
Methodological framework for developing a proxy indicator of Child Criminal Exploitation risk based on socio-spatial stressors (Agnew, 1992, 2013).

This study aimed to develop a proxy indicator for identifying areas in NI where the risk of CCE may be elevated due to the convergence of multiple socio-spatial stressors. Drawing on General Strain Theory (Agnew, 1992, 2013), the design integrates criminological theory with spatial analysis to assess how contextual harms—education, deprivation, violence and paramilitary activity—cluster geographically. Parametric analysis of paramilitary activity was conducted at both the ward and Local Government District (LGD) levels. By operationalising these conditions into composite search criteria, we were able to isolate a reduced number of wards for further scrutiny (Figure 1).

### 2.2. Contextual Strain Indicators

Seven publicly available datasets were selected to operationalise the five contextual strains, and these indicators were aggregated to identify areas where multiple risk factors intersect: (1) educational attainment—percentage of residents aged 16 and over with no qualifications; (2) youth density—percentage of residents in the age band 5 to 19 years old; (3) violent crime prevalence—percentage of all crime that is violent crime (including drug incidents, possession of weapons, and violent and sexual offences); (4) deprivation—Multiple Deprivation Rank where 1 is the most deprived area and 462 is the least deprived area; (5) paramilitary activity—(a) casualties as a result of paramilitary-style shootings 2022–2024 by LGD; (b) casualties as a result of paramilitary-style assaults from 2022 to 2024 by LGD; (c) paramilitary attacks from 10 April 1999 to 14 February 2023 by electoral ward.

### 2.3. Data Sources and Pre-Processing

A total of 354,528 street-level crime records from May 2021 to July 2023 (Data.police.uk, 2024) along with demographic data from the 2021 Census (NISRA, 2017, 2023, 2024) were incorporated into a geodatabase. Age-related data were provided in five-year bands (0–4 to 90+); for this study, the 5–19 age group was selected as most relevant to CCE vulnerability. Violent crime was operationalised using PSNI categories (PSNI, 2021), including drug offences, weapon possession, and violence/sexual offences, totalling 136,781 incidents. These were aggregated at the ward level to form a key strain indicator of the spatial distribution of violent crime.

### 2.4. Spatial Analysis

Geospatial processing was conducted using Esri’s software, including ArcGIS Pro v3.4.2 and the Data Interoperability extension (ESRI, 2024a) for automated ETL workflows. Postcode-level crime data were geocoded (ESRI, 2024b) and spatially joined with ward-level demographic and deprivation indicators, enabling the identification of high-risk zones. Once geocoded, point data were spatially aggregated using polygon feature layers representing electoral wards and Local Government Districts (LGDs). The Intersect tool was applied to map point-level crime data to ward boundaries, allowing for the calculation of crime totals per ward. Ward-level aggregation was selected to balance granularity with ethical considerations, given the sensitivity of CCE-related data. Distance bands were optimised to ensure robust spatial clustering, and incremental spatial autocorrelation was used to refine the analysis. The final proxy model reduced the initial 462 wards to a smaller subset, enabling more targeted and efficient resource allocation.

### 2.5. Proxy Development

Four search criteria were developed by combining thresholds across seven indicators. These criteria progressively narrowed the geographic scope, identifying wards with elevated contextual strain. The model was designed to be scalable and adaptable to other jurisdictions using commonly available census and crime data.

### 2.6. Statistical Analysis

#### 2.6.1. Spatial Statistics—Hot Spot and Cluster Outlier Analysis

Hot spot and cluster outlier analyses were conducted to evaluate the null hypotheses that the spatial distribution of violent crime in NI is the result of a random spatial process. The analyses focused on the following categories: (a) total count of violent crimes per ward; (b) overall crime rate per 1000 population; (c) violent crime rate per 1000 population; (d) percentage of total crime that is violent crime in each ward; and (e) areas where people are disproportionately exposed to violent crime.

These analyses provided insights into the spatial patterns of violent crime in NI and informed the development input parameters to the CCE proxy. This paper presents findings specifically related to the violent crime rate per 1000 population and the percentage of total crime that is violent crime in each ward.

Hot spot analysis was employed to identify statistically significant clusters of high and low values for both indicators. This method assesses the likelihood that observed spatial patterns are due to random chance. Using the Getis-Ord Gi* statistic (Getis & Ord, 1992; Lloyd, 2011; Fotheringham, 2009), the analysis identifies statistically significant hot spots (clusters of high values) and cold spots (clusters of low values), thereby supporting evidence-based resource allocation rather than decisions based on random spatial variation.

Optimised hot spot analysis was used to determine the appropriate neighbourhood scale for analysis (see Table 1). This tool identifies statistically significant spatial clusters and adjusts for multiple testing and spatial dependence. It serves as a robust starting point by interrogating the data to produce optimal settings, which can then be refined by the user using the Hot Spot Analysis tool (ESRI, 2025).

#### 2.6.2. Cluster and Outlier Analysis (Anselin Local Moran’s I)

Cluster and outlier analysis using Anselin Local Moran’s I (Anselin, 1995; Lloyd, 2011; Rogerson, 2010) was employed to identify spatial clusters of features with high or low values as well as spatial outliers. In this analysis, each feature is excluded from its own neighbourhood, allowing for independent assessment. Two key questions guide the interpretation: (1) Is the neighbourhood significantly different from all other neighbourhoods? (2) Is the feature significantly different from all other features?

The output categorises features into the following cluster types: (a) high–high (HH); (b) high–low (HL); (c) low–low (LL); and (d) low–high (LH). These classifications are grounded in Tobler’s First Law of Geography (Tobler, 1970). Deviations from expected spatial similarity—such as HL and LH—are outliers and represent spatial association of dissimilar values. Outliers are unlikely to be random and warrant further investigation.

#### 2.6.3. Spatial Statistics—Distance Band for Analysis

Selecting an appropriate distance band is a critical component of spatial analysis, particularly for accurate detection of clusters and outliers. In this study, optimised hot spot analysis was employed alongside a review of the spatial characteristics of electoral wards in NI to determine suitable distance thresholds. This tool provides an initial overview of spatial clustering, which can be interpreted in relation to the research question. Where appropriate, alternative distance bands may be manually specified to better reflect the underlying data, e.g., England or other areas.

To refine the selection of distance bands, incremental spatial autocorrelation was applied. Statistically significant clusters of violent crime were used to inform the development of search criteria for a proxy measure of contextual predictors of CCE. Ward-level data associated with these clusters were extracted and analysed to identify common characteristics. These characteristics were incorporated into the strain proxy, supporting the hypothesis that spatial clusters of violent crime may serve as a proxy for identifying areas at heightened risk of CCE as discussed in the results.

## 3. Results

### 3.1. Distance Band Outcomes Following Analysis

Spatial analysis revealed significant variation in ward size and shape across Northern Ireland. Optimal distance bands for detecting violent crime clusters were identified using incremental spatial autocorrelation, with 20,287.30 m selected for violent crime rate per 1000 population and 13,927 m for the proportion of all crime that is violent crime (Table 1). These thresholds ensured robust clustering, with all 462 wards having sufficient spatial neighbours or analysis. Additional distances were also reviewed; see Appendix A.

Hot spot and cluster outlier analysis confirmed that violent crime is not randomly distributed, allowing the null hypothesis to be rejected. These findings informed the development of a proxy model for identifying areas at elevated risk of CCE, based on statistically significant patterns.

**Figure 2:**
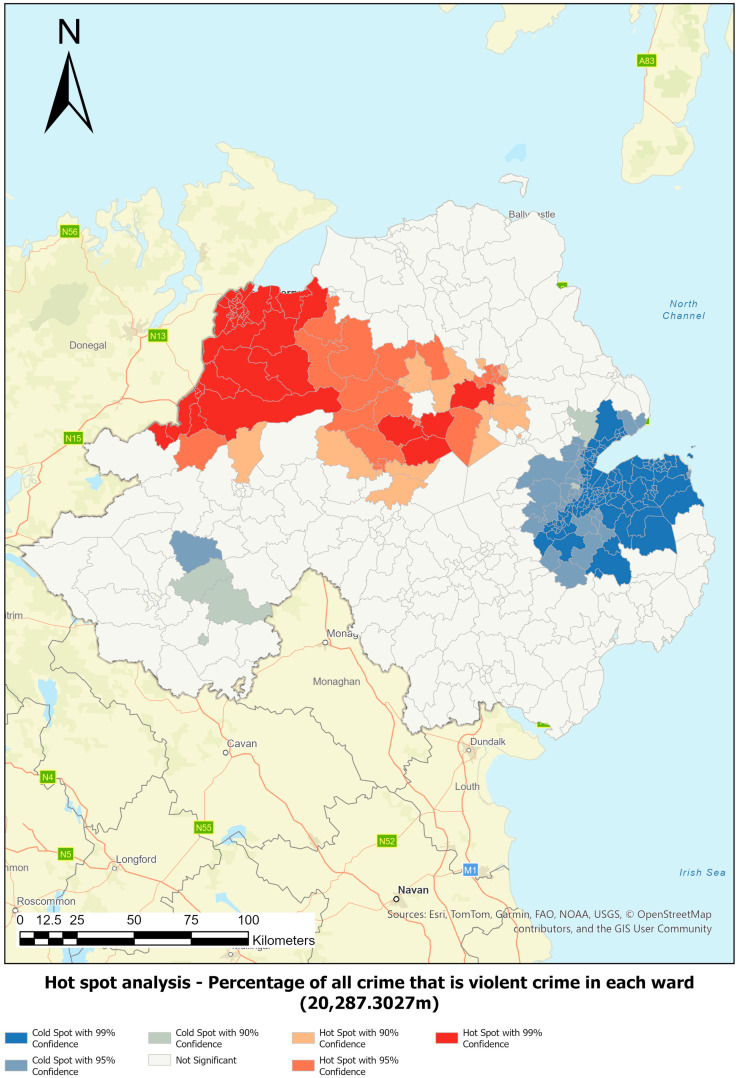
Hot spot analysis output for percentage of total crime that is violent crime in each ward at a distance band of 20,287.3027 m.

**Figure 3:**
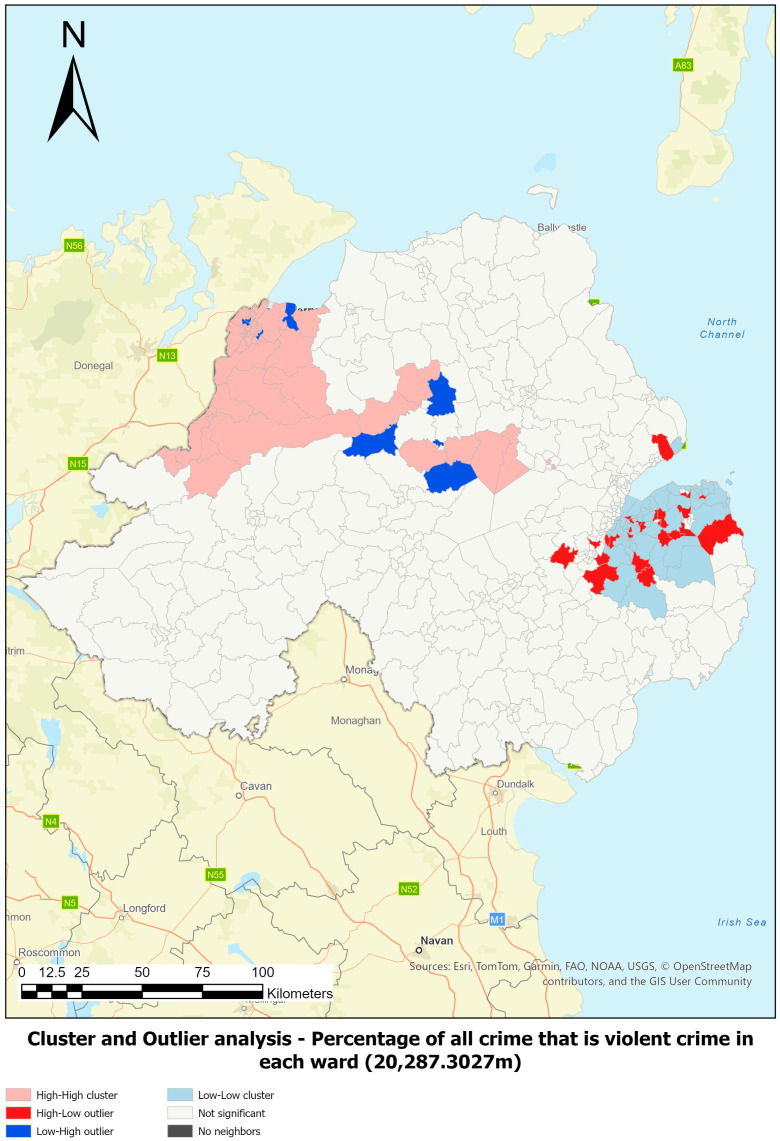
Cluster and outlier analysis for percentage of total crime that is violent crime in each ward at a distance band of 20,287.3027 m.

Figure 2 and Figure 3 illustrate the results of the hot spot and cluster outlier analysis for the percentage of total crime that is violent crime. Table 2 outlines the input threshold values that were representative of the conditions observed in statistically significant cluster areas.

### 3.2. Identification of Statistically Significant Clusters and Proxy Development

Across Northern Ireland, between 39 and 255 statistically significant clusters of violent crime were identified depending on the analytical parameters applied (Table 1). Following the initial identification of 76 wards under Search Criteria One, further refinement was achieved through the development of Search Criteria Two, which reduced the number identified to 45 wards. Table 3 presents summary statistics for the wards identified in Search Criteria One wards, highlighting key indicators such as deprivation and educational attainment. When compared to the full NI dataset (Table 3), these wards exhibit distinct socio-demographic profiles, supporting their prioritisation in CCE-related exploratory work. This reduction from the original 462 electoral wards demonstrates the utility of spatial filtering in narrowing the investigative scope. The criteria used were based on violent crime clustering and associated socio-demographic indicators.

The value of this spatial focus aligns with existing research showing that violence remains a persistent, embedded and enduring feature of everyday life for young people in certain communities (Hamill, 2011; Walsh, 2021, 2023). Paramilitary organisations do not operate outside the community structure; rather, they are interwoven into local social fabrics (Walsh, 2021). In such contexts, violence is often normalised, enacted both among peers and by adults associated with paramilitary groups, and is closely linked to entrenched forms of coercive control (Hamill, 2011). CCE emerges as a specific manifestation of this wider landscape of violence, wherein young people’s involvement in criminality may be directed, facilitated, or coerced by others, with significant implications for their safety and wellbeing (Walsh, 2023).

Against this backdrop, the identification and review of statistically significant areas where violence levels exceed those of surrounding locations constitutes an appropriate and methodologically defensible starting point for this scoping analysis. By isolating spatial concentrations of violence and linking these to relevant socio-demographic proxies, the analysis enhances the capacity to identify localities where the structural conditions for CCE may be particularly acute. Additionally, as research in this field progresses, insights emerging from organisations and frontline practitioners (Walsh et al., 2025b) will further refine the database and strengthen subsequent analytical outputs. The database can therefore be iteratively supplemented and interrogated as new data, intelligence, and learning become available.

### 3.3. Refinement of Search Criteria and Integration of Additional Data

Subsequent to the initial identification analysis, additional datasets became available, enabling the creation of Search Criteria Three and Four (PSNI, 2025). These criteria incorporated ward-level data on paramilitary and organised crime group (OCG) activity, offering a finer spatial resolution than previously available LGD data. The integration of this new information allowed for more precise identification of wards exhibiting multiple contextual strains relevant to CCE.

Across all four search criteria, the geographic and demographic scope was significantly reduced: the land area under investigation decreased from 100% of NI to between 14.45% and 0.84%; the population affected (i.e., the number of residents that would require investigation) decreased from 100% (all of NI) to 17.91% to 1.41%. These reductions illustrate the practical utility of the proxy model in guiding targeted interventions. The refined search areas are not only statistically significant but also socio-demographically distinct, as evidenced by comparisons within Table 3 (Search Criteria One and NI-wide summary statistics). Wards identified under Criteria One show higher levels of deprivation and lower educational attainment than the NI average, reinforcing their relevance for focused investigation. The interrogation of the geospatial database enabled extraction of ward-level intelligence. The finer-grained insights allow for targeted prioritisation of resources based on policy needs, such as educational disadvantage or deprivation.

By integrating multiple public datasets and refining spatial granularity from Local Government Districts to electoral wards, the method supports iterative model development responsive to emerging data. Four predictive models were developed to highlight wards where contextual strains—such as deprivation, paramilitary activity, and youth vulnerability—converge. This GIS-based framework not only enhances operational decision-making but also provides a scalable foundation for future research and intervention strategies.

## 4. Discussion

This study demonstrates the utility of geostatistical methods in identifying areas at elevated risk of CCE. The integration of multiple public datasets covering the five contextual strains enabled the development of a proxy model that spatially isolates high-risk wards and allows for the examination of socio-demographic characteristics at the ward level.

The results confirm that violent crime is not randomly distributed across NI but instead clusters in areas exhibiting overlapping socio-demographic vulnerabilities. The refined search criteria significantly reduced the geographic scope by 99% and the population by 98.59%, supporting efficient resource allocation and demonstrating the potential for data-driven prioritisation in operational contexts. The value of this approach is compounded by the additional information available for these areas and contained within the created geodatabase, incorporating additional census data variables, allowing for further investigation, i.e., the database that has been created is not limited to the five contextual strains presented.

Kane and Chisholm (2025, p. 41) state that “interview data indicates an awareness of CCE as a phenomenon that has occurred for decades in NI without formal recognition despite legal and policy frameworks and vast support structures relating to child protection”. While the context in which this study was undertaken illustrates the limitations when data are limited, it also illustrates the utility of proxy indicators. These represent a valuable advancement in identifying areas where there is an elevated risk of CCE. For instance, in areas of conflict and/or high organised crime, potential victims may be more unwilling or too fearful to disclose harmful experiences at home or within their community. Van Der Watt (2019, p. 765) details similar issues in relation to human trafficking, citing Holmes (2010), where victims themselves often do not consider reporting due to the “perceived, and sometimes real, disincentive for approaching the authorities”. GISs can assist practitioners by highlighting risk levels within specific locations. This enables context-sensitive responses—such as categorising areas as low, medium or high alert—based on flagged indicators.

Importantly, this approach recognises that CCE operates within a complex adaptive system, where multiple interfacing factors—social, spatial and structural—shape risk patterns. Understanding these dynamics is critical for developing responsive, evidence-based interventions that adapt to evolving contexts rather than relying solely on static datasets. Given the embedded nature of CCE within community structures, a mechanism was required to review where it is most likely to occur. A substantial body of sociological and criminological research demonstrates that children involved in offending behaviour have frequently experienced victimisation themselves. Violence, in particular, is one of the most common forms of harm affecting children and young people (Baglivio & Wolff, 2021). Global estimates from the World Health Organization (World Health Organization [WHO] et al., 2020) underscore this burden, with estimates that around one out of every two children aged 2–17 years suffer some form of violence annually, with one-third of adolescents victimised by peer-related violence (World Health Organization [WHO] et al., 2020). Hillis et al. (2016) similarly estimate that up to one billion children are affected annually. Patterns of exposure vary by age: younger children are more likely to be harmed within the family environment (Bellis et al., 2017), whereas adolescents experience elevated exposure to community-level violence (Walsh et al., 2025a). From a policy perspective, these findings offer a scalable framework for identifying communities where contextual strains converge. This supports practitioners tailoring interventions to local conditions, particularly in areas characterised by high deprivation, low educational attainment and other indicators associated with heightened vulnerability. By integrating spatial analysis with socio-demographic insights, decision-makers can more effectively prioritise regions where structural conditions of CCE are most acute and where preventative or early-intervention approaches have greatest impact.

The study contributes methodologically by demonstrating how GISs can be used to operationalise criminological perspectives on CCE within spatial contexts. By conceptualising contextual harms as geographically embedded strains, the research advances place-based extension of strain theory. The analytical approach enabled results to be reviewed at a macro-, meso-, and micro-spatial scales, allowing for differentiated interventions depending on the context. Whilst criminological studies frequently isolate conditioning variables, this spatial contextual approach has allowed for the broader environmental context in which strain occurs to be considered and explored.

## 5. Limitations and Directions for Future Research

This study utilised street-level crime data from 2021 to 2023. Interpretations based on these data must account for the time lag between data collection, analysis, interpretation and implementation. A key advantage of the GIS-based approach is its capacity to rapidly process and interrogate large datasets, making it adaptable for future iterations as new data become available.

The primary aim was to demonstrate the utility of the proposed methodology in identifying areas where conditions may exacerbate the risk of CCE. Further research will involve multi-agency collaboration and police data to validate whether the identified locations correspond with actual CCE activity—a “ground-truthing” process is essential for refining the model and ensuring its relevance across diverse geographical contexts. A defining feature of CCE is spatial displacement: recruitment may occur in one location, while coerced criminal activity takes place elsewhere (Norman, 2024). Such mobility reflects the dynamic nature of CCE within a complex adaptive system where social, spatial, and structural factors interact fluidly (Van Der Watt, 2019; Lowe et al., 2023). Static spatial models risk oversimplifying these dynamics; thus, this study represents an initial step toward developing spatial indicators that can be utilised to explore linking recruitment and activity sites and using multi-source data to refine predictive accuracy. Although NI served as the case study, the methodological framework has global applicability. By adapting the model to local datasets, researchers and practitioners can examine geographic patterns of CCE elsewhere. Identifying spatial clusters of risk enables targeted, multi-agency interventions to support vulnerable young people.

Future research will focus on validating the proxy through collaborative efforts and incorporating real-time and temporal data. The inclusion of additional indicators—such as school exclusions, housing instability and access to youth services—will enhance both the predictive accuracy and practical utility. Ongoing engagement with practitioners and policymakers will be critical to ensure that the model remains actionable and ethically grounded.

## 6. Conclusions

This study demonstrates the value of spatial analysis in understanding and responding to contextual risks associated with CCE. By integrating criminological theory with geostatistical methods leveraging GIS and publicly available datasets, the research advances proactive, evidence-based strategies for safeguarding vulnerable populations. CCE is increasingly recognised as a complex and under-acknowledged form of child abuse, involving manipulation, coercion, and control of children for criminal gain. While this phenomenon has gained policy traction in other parts of the UK and Ireland, NI presents a unique socio-political context marked by conflict, paramilitary activity, higher levels of deprivation, and systemic inequalities.

General Strain Theory (Agnew, 1992) provides a useful lens for understanding how contextual strains contribute to the emergence of CCE. GST posits that strain—arising from blocked opportunities, loss of valued goals, or exposure to adverse stimuli—creates pressure that may lead individuals to adopt illegitimate coping strategies when legitimate options are available. In the NI context, strains such as deprivation, exposure to paramilitary violence, and exclusion compound individual vulnerabilities. These conditions heighten psychological stress and normalise coercion and criminality within local communities, reducing resistance to exploitation. When combined with limited access to protective resources, these strains create an environment where recruitment into criminal activity becomes a perceived coping mechanism. Conversely, mitigating factors—such as strong social support or community-based interventions—can disrupt this causal pathway by providing legitimate alternatives to strain-induced pressures. Understanding these mechanisms is critical for designing interventions that address both structural harms and individual coping capacities.

Findings support the hypothesis that community-level factors elevate CCE risk. While the methodology does not identify individual cases, it highlights areas where CCE is likely to cluster—typically characterised by higher youth populations, low educational engagement, elevated violence-related crime, paramilitary activity, and socioeconomic deprivation. Geospatial analysis using proxy indicators of contextual strain revealed clear clustering of CCE risk factors at both ward and LGD levels. Street-level crime data provided further insight into prevalent criminal activities, informing the development of tailored intervention strategies.

The analysis offers practical applications in three key areas: (a) predictive modelling to identify potential CCE clusters, with future validation planned using retrospective police data and prospective crime reporting; (b) resource allocation, enabling targeted interventions tailored to high-risk communities; (c) policy testing, to assess which contextual domains are most amenable to change and effective in reducing CCE.

Future research will refine the approach through ground-truthing and weighting of individual strain factors to enhance predictive validity.

## Figures and Tables

**Table 1:** Outcome of hot spot analysis data—statistically significant clusters.

Violent Crime Criteria	Violent Crime Rate/1000	Violent Crime Rate/1000	Violent Crime Rate/1000	% of All Crime That Is Violent Crime (Ward)	% of All Crime That Is Violent Crime (Ward)	% of All Crime That Is Violent Crime (Ward)
Neighbourhood distance (metres)	16,093.40	20,287.30	32,186.90	13,927.00	16,093.40	20,287.30
Valid input features	462	462	462	462	462	462
Outliers	1	1	1	1	1	1
Statistically significant clusters of high and low violent crime rate (by ward per 1000 and percentage of all crime that is violent crime accordingly) based on an FDR correction for multiple testing and spatial dependence	136	52	39	203	217	255
Percentage of features with less than 8 neighbours	3.9	0.9	0	7.8	3.9	0.9

**Table 2:** Contextual risk predictors, search parameters and search criteria for CCE methodological exploration.

Contextual Risk Predictors	Search Parameter	Search Criteria One	Search Criteria Two	Search Criteria Three	Search Criteria Four
Educational attainment	No qualifications	20% or more	25% or more	20% or more	25% or more
Youth density	Age band 5–19	15% or more	15% or more	15% or more	15% or more
Paramilitary/ OCG activity	Casualties as a result of paramilitary-style assaults	More than 1 assault by LGD	More than 1 assault by LGD	More than 1 assault by ward	More than 1 assault by ward
	Casualties as a result of paramilitary-style shootings	More than 3 shootings by LGD	More than 3 shootings by LGD	More than 1 shooting by ward	More than 1 shooting by ward
Deprivation	Multiple Deprivation Measure rank	Rank 200 or less	Rank 100 or less	Rank 200 or less	Rank 100 or less
Violent crime prevalence	Percentage of all crime that is violent crime in that ward	35% or more	35% or more	35% or more	35% or more
	**Total number of wards (out of 462) that match these criteria**	76 wards	45 wards	14 wards	5 wards
	**Area in m^2^**	1,957,900,489.0	846,924,242.5	471,357,797.2	113,287,515.2
	**Number of residents**	340,783	207,658	62,474	26,772
	**Area percentage compared to all of NI**	14.45	6.25	3.48	0.84
	**Residents percentage compared to all of NI**	17.91	10.91	3.28	1.41

**Table 3:** Comparison of summary statistics for Search Criteria One contextual risk predictors compared to the geographic region of Northern Ireland.

		Percentage of All Crime That Is Violent Crime in That Ward	Percentage of Population in Ward with No Qualifications	Multiple Deprivation Measure Rank	Percentage Age Band 5 to 19	Violent Crime Rate per 100,000 by Ward	Casualties as a Result of Paramilitary-Style Shootings 2022–2024 (LGD)	Casualties as a Result of Paramilitary-Style Assaults 2022–2024 (LGD)	Paramilitary Attacks from 10 April 1999 to 14 February 2023 (Ward)
Seach Criteria One summary statistics for contextual risk predictors	Min	35.9	20.5	1	15.7	2703.5	3	5	/
	Max	54.5	45.5	193	28.8	29,431.5	10	23	/
	Mean	43.5	30.8	80	20.3	9255.2	7	12	/
Summary statistics for the geographic region of Northern Ireland	Min	20.8	4.8	1	9.6	512.4	0	0	1
	Max	60.9	46.0	462	28.8	76,218.4	10	23	600
	Mean	39.6	24.0	232	19.0	6584.5	2	6	59

## Data Availability

No new data were created or analysed in this study.

## Appendix A. Distance Band Outcomes Following Analysis—Size and Shape of Northern Ireland

Analysis of the spatial characteristics of wards revealed substantial variation in both size and shape:

- Area (m^2^): minimum = 536,427.46; maximum = 256,896,846.20; mean = 30,985,463.80 m^2^.
- Length (m): minimum = 3127.30; maximum = 133,480.06; mean = 26,297.36.

Additional distances—13,927.00 m, 16,093.40 m, 20,287.30 m and 32,186.90 m—were reviewed to assess clustering intensity. A total of 462 valid input features (electoral wards) were included in the analysis. For violent crime rate per 1000 population, the descriptive statistics were minimum = 5.12, maximum = 762.18, mean = 65.84 and standard deviation = 61.00. One outlier location was excluded from the computation of the optimal fixed distance band. The final selection of 32,186.90 metres was based on clustering intensity and ensured 100% of features had at least eight neighbours—an important criterion for robust spatial analysis.

- For the percentage of all crime that is violent crime, the descriptive statistics were minimum = 20.79%, maximum = 60.87%, mean = 39.64% and standard deviation = 6.11%.
